# RNAi-mediated silencing of CD147 inhibits tumor cell proliferation, invasion and increases chemosensitivity to cisplatin in SGC7901 cells *in vitro*

**DOI:** 10.1186/1756-9966-29-61

**Published:** 2010-06-03

**Authors:** Bo Wang, Yong-Fei Xu, Bang-Shun He, Yu-Qin Pan, Li-Rong Zhang, Chan Zhu, Li-Li Qu, Shu-Kui Wang

**Affiliations:** 1 Department of Life Sciences, Nanjing Normal University, Nanjing, Jiangsu 210046, China; 2 Central Laboratory of Nanjing First Hospital Affiliated to Nanjing Medical University, Nanjing, Jiangsu 210012, China

## Abstract

**Background:**

CD147 is a widely distributed cell surface glycoprotein that belongs to the Ig superfamily. CD147 has been implicated in numerous physiological and pathological activities. Enriched on the surface of many tumor cells, CD147 promotes tumor growth, invasion, metastasis and angiogenesis and confers resistance to some chemotherapeutic drugs. In this study, we investigated the possible role of CD147 in the progression of gastric cancer.

**Methods:**

Short hairpin RNA (shRNA) expressing vectors targeting CD147 were constructed and transfected into human gastric cancer cells SGC7901 and CD147 expression was monitored by quantitative realtime RT-PCR and Western blot. Cell proliferation, the activities of MMP-2 and MMP-9, the invasive potential and chemosensitivity to cisplatin of SGC7901 cells were determined by MTT, gelatin zymography, Transwell invasion assay and MTT, respectively.

**Results:**

Down-regulation of CD147 by RNAi approach led to decreased cell proliferation, MMP-2 and MMP-9 activities and invasive potential of SGC7901 cells as well as increased chemosensitivity to cisplatin.

**Conclusion:**

CD147 involves in proliferation, invasion and chemosensitivity of human gastric cancer cell line SGC7901, indicating that CD147 may be a promising therapeutic target for gastric cancer.

## Background

Although the incidence and mortality of gastric cancer have fallen dramatically over the past 50 years [1], it remains the fourth most common cancer and the second leading cause of cancer-related death worldwide [2,3]. Gastric cancer traditionally carries a very poor prognosis because of late presentation at an advanced stage of disease and remains a great clinical challenge. Therefore, a better understanding of the molecular mechanisms underlying gastric cancer formation and progression should be helpful in developing more effective treatments for this disease.

The metastatic process is dependent on the degradation of the extracellular matrix (ECM) both at primary tumor site and at secondary colonization site. Matrix metalloproteinases (MMPs), a family of zinc-dependent proteolytic enzymes, play a central role in the degradative process. High levels of MMPs have been frequently found at the tumor-stroma interface, most of which are expressed by stromal cells rather than by tumor cells themselves [4]. A search for MMP inducing factors in tumor cells led to the identification of CD147/EMMPRIN [5]. CD147 is a highly glycosylated cell surface transmembrane protein which is expressed at high levels in variety of malignant human cancers. In cells, CD147 is expressed in various forms, including high glycosylated (HG 45-65 kDa) and low glycosylated (LG 32-44 kDa) forms as well as the native 27-kDa protein. CD147 has been demonstrated to stimulate production of MMP-1, -2, -3, -9, -14, and -15 in peritumoral fibroblasts and endothelial cells therefore facilitate tumor invasion and metastasis [6]. Recently, CD147 was found to stimulate tumor angiogenesis by elevating vascular endothelial growth factor (VEGF) and MMP expression in neighboring fibroblasts via the PI3K-AKT signaling pathway [7,8]. CD147 is also involved in multidrug resistance of cancer cells via hyaluronan-mediated activating of ErbB2 signaling and cell survival pathway activities [9-11].

Zheng et al. [12] investigated the role of CD147 in progression and angiogenesis of gastric cancer. CD147 expression was gradually increased from normal mucosa to carcinomas through hyperplastic or metaplastic mucosa of the stomach, and its expression was positively correlated with tumor size, depth of invasion, lymphatic invasion and expression of ki-67, MMP-2, MMP-9 and VEGF in gastric cancer. However, the effect of reducing CD147 levels by genetic methods in established gastric cancer cells has not been investigated, the study of which would help understand its role in the malignant phenotype. Therefore, in this study, we silenced CD147 expression in human gastric cancer cell line SGC7901 by RNA interference (RNAi) to determine its effect on the proliferation and invasion ability as well as the chemosensitivity of SGC7901 cells.

## Methods

### Cell culture

Human gastric cancer cell line SGC7901 was provided by Digestive Department of Jiangsu Province Hospital, China. Cells were cultured with DMEM medium (Gibco BRL, Grand Island, NY, USA) supplemented with 10% newborn calf serum (Gibco BRL, Grand Island, NY, USA) at 37°C in a humidified atmosphere containing 5% CO_2_.

### Construction of shRNA expression vectors

The vector pSilencer 3.1-H1 neo (Ambion Inc., Austin, TX, USA) was used to generate short hairpin RNA (shRNA) specific for CD147. Two different regions of CD147 mRNA [GenBank: AB085790] were selected as the RNAi target sites: 370-390 bp and 808-828 bp [13]. Two pairs of template oligonucleotides, each encoding one of the target sequences were designed and synthesized (designated as shRNA1 and shRNA2 respectively), and another pair of oligonucleotides (designated as shRNA-control) encoding a non-specific shRNA used as a negative control was also synthesized (Table 1). These oligonucleotides were annealed and subcloned into the *Hin*d III and *Bam*H I sites of the vector according to the manufacturer's instructions. These recombinant vectors were designated as pSilencer-shRNA1, pSilencer-shRNA2 and pSilencer-shRNA-control, respectively. They were sequenced for correct ligation.

**Table 1:** The sequences of the designed CD147 specific shRNAs

shRNA	Sequence
shRNA1	5'-GATCCGTCGTCAGAACACATCAACTTCAAGAGAGTTGATGTGTTCTGACGACTTTTTTGGAAA-3'
	5'-AGCTTTTCCAAAAAAGTCGTCAGAACACATCAACTCTCTTGAAGTTGATGTGTTCTGACGACG-3'
shRNA2	5'-GATCCGTGACAAAGGCAAGAACGTCTTCAAGAGAGACGTTCTTGCCTTTGTCATTTTTTGGAAA-3'
	5'-AGCTTTTCCAAAAAATGACAAAGGCAAGAACGTCTCTCTTGAAGACGTTCTTGCCTTTGTCACG-3'
shRNA-control	5'-GATCCACTACCGTTGTTATAGGTGTTCAAGAGACACCTATAACAACGGTAGTTTTTTTGGAAA-3'
	5'-AGCTTTTCCAAAAAAACTACCGTTGTTATAGGTGTCTCTTGAACACCTATAACAACGGTAGTG-3'

### Transfection of cells

SGC7901 cells were plated in six-well plates at a density of 3 × 10^5 ^cells per well and incubated overnight. Cells were transfected with pSilencer-shRNA1, pSilencer-shRNA2 and pSilencer-shRNA-control respectively using Lipofectamine 2000 (Invitrogen, Carlsbad, CA, USA) according to the manufacturer's instructions. Forty-eight hours after transfection, SGC7901 cells were diluted to 1:10 for passage and neomycin resistance clones were selected in the medium containing 400 μg/ml G418 (Gibco BRL, Grand Island, NY, USA) for two weeks. The positive clones were picked and expanded to establish cell lines. The stable transfection cell clones, designated as SGC7901/shRNA1, SGC7901/shRNA2 and SGC7901/shRNA-control, were verified by quantitative realtime RT-PCR and Western blot analysis.

### Quantitative Realtime RT-PCR Assays

Total cellular RNA was extracted using TRIzol reagent (Invitrogen, Carlsbad, CA, USA). RNA integrity was confirmed by electrophoresis on ethidium bromide-stained 1% agarose gel. The primer sequences used were for CD147:(sense)5'-CCATGCTGGTCTGCAAGTCAG-3' and(antisense) 5'-CCGTTCATGAGGGCCTTGTC-3'; β-actin(sense)5'-CTGGAACGGTGAAGGTGACA-3' and (antisense) 5'-AAGGGACTTCCTGTAACAACGCA-3'. The mRNA level for CD147 was analyzed by one-step realtime reverse transcriptase polymerase chain reaction with RNA-direct™ SYBR Green Realtime PCR Master Mix (Toyobo Co., Ltd., Osaka, Japan) according to the manufacturer's instructions. Cycling conditions were: 90°C for 30 s, 61°C for 20 min, 95°C for 60 s, then 40 cycles at 95°C for 15 s, 60°C for 1 min. The mRNA level for CD147 of each sample was normalized to that of the β-actin mRNA and presented as unit values of 2^ [Ct_(β-actin) _- Ct_(CD147)_]. The amplification was monitored on an ABI prism 7500 realtime PCR apparatus (Applied Biosystems, USA).

### Western blot analysis

Cells were harvested from flasks, and lysed with ice-cold lysis buffer (50 mM Tris-HCl, pH 7.4, 150 mM NaCl, 1 mM MgCl_2_, 100 μg/ml PMSF and 1% Triton X-100) for 30 min on ice. Cell lysates were then collected after centrifugation at 12,000 rpm for 5 min at 4°C. Equal amounts (50 μg) of lysate proteins were separated on 10% SDS-PAGE gels, and transblotted onto PVDF membranes (Pall Corporation, USA). After blocking with 5% non-fat dry milk in TBST buffer (10 mM Tris, pH 7.5, 150 mM NaCl, and 0.05% Tween 20) for 2 h at room temperature, the membranes were probed with 1:500 dilution of anti-CD147 (Santa Cruz, CA, USA) or anti-β-actin (Santa Cruz, CA, USA) antibodies at room temperature for 2 h, followed by incubation in a 1:2000 dilution of secondary antibodies conjugated to horseradish peroxidase (Santa Cruz, CA, USA) for 1 h at room temperature. Protein bands were detected using ECL detection system (Boster, Wuhan, China). All of the Western blots were performed at least three times.

### Cell Proliferation Assay

Before the cell proliferation assay, trypan blue exclusion test of cell viability was performed and the viability of the three groups of cells (SGC7901, SGC7901/shRNA-control and SGC7901/shRNA2) was >98%. Cell proliferation *in vitro *was analyzed with 3-(4, 5-dimethylthiazol-2-yl)-2, 5-diphenyltetrazolium bromide (MTT, Sigma, St. Louis, MO, USA). Briefly, 2000 cells of each group were plated per well in three 96-well microplates in 200 μL of medium. After 24, 48, and 72 h of culture respectively, 20 μL of MTT substrate (5 mg/mL in PBS) was added to each well, and the plates were returned to standard tissue incubator conditions for an additional 6 h. The medium was then removed, the cells were solubilized in 150 μl of dimethyl sulfoxide, and colorimetric analysis was performed (wavelength, 490 nm). The inhibition rate was calculated as [1 - (OD value of the transfectant/OD value of untreated SGC7901)] × 100%. Each experiment was done in triplicate.

### Gelatin zymography

Protein concentrations in conditioned medium were determined using the bicinchonic acid method (BCA kit) (Pierce, Rockford, IL, USA). The gelatinolytic activities of MMP-2 and MMP-9 in the conditioned medium were assayed by electrophoresis on 10% polyacrylamide gels containing 1 mg/ml of gelatin (type A, Sigma, St. Louis, MO, USA) at 4°C. PAGE gels were run at 120 V, washed in 2.5% Triton X-100 for 1 h, and then incubated for 20 h at 37°C in activation buffer (50 mM Tris-HCl, pH 7.5, 5 mM CaCl_2_, 0.02% Brij-35). After staining with Coomassie Blue (10% glacial acetic acid, 30% methanol and 0.5% Coomassie Blue) for 3 h, the gel was destained with a solution of 10% glacial acetic acid, and 50% methanol without Coomassie Blue for 1 h. White lysis zones indicating gelatin degradation were revealed by staining with Coomassie blue R-250.

### Invasion assay

Appropriate Matrigel (BD Biosciences, Bedford, MA, USA)was added to the upper chamber of the transwell apparatus with 8-μm pore size membrane (Costar, Cambridge, MA, USA). After the Matrigel solidified at 37°C, serum-free DMEM containing 1 × 10^5 ^cells in 100 μl was added into the upper chamber; the lower chamber received 500 μl of 10% FBS-containing medium. After incubated at 37°C for 24 h, membranes coated with Matrigel were swabbed with a cotton swab and fixed with 100% methanol for 10 min. The membranes with cells were soaked in 0.1% crystal violet for 10 min and then washed with distilled water. The number of cells attached to the lower surface of the polycarbonate filter was counted at 400× magnification under a light microscope. Results were expressed as mean of triplicate experiments.

### Drug sensitivity assay

To assess the chemosensitivity to anti-tumor drug cisplatin, the cells were seeded in triplicate on 96-well plates at 1 × 10^4 ^cells/well and incubated for 24 h. The medium was then removed and replaced with fresh medium containing cisplatin (Sigma, St. Louis, MO, USA) with varying concentrations: 0.1 × peak plasma concentration (PPC), 1 × PPC and 10 × PPC. After 48 h, cells were treated with MTT as described earlier. The inhibition rate was calculated as [1 - OD_490(cisplatin+)_/OD_490(cisplatin-)_] × 100%. The assay was repeated three times.

### Statistical analysis

SPSS13.0 software was used. Each assay was performed at least three times. The data were expressed as mean ± SD, and Student's *t *test was used to determine the significance of differences in multiple comparisons. p < 0.05 was considered to be statistically significant.

## Results

### shRNA targeting CD147 suppresses CD147 expression in SGC7901 cells

**Figure 1:**
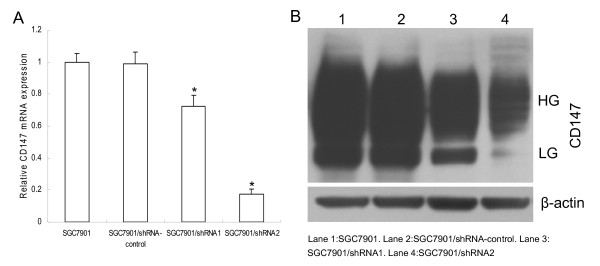
**CD147 specific shRNAs results in the reduction of CD147 mRNA and protein levels in SGC7901 cells**. (A). Relative mRNA levels were analysed by quantitative RT-PCR. β-actin was used as normalization control. *p < 0.01 compared with SGC7901. (B). Western blot analysis of CD147 protein expressions. β-actin was used as loading control. HG:high glycosylated form; LG: low glycosylated form.

The silencing effects of different CD147 specific shRNAs in SGC7901 cells were first evaluated using realtime RT-PCR. A 27.6% and an 82.7% CD147 mRNA inhibition for shRNA1 and shRNA2 was achieved respectively compared to untreated SGC7901 cells (Fig. 1A), while shRNA-control showed no effects. Western blot analysis confirmed the down-regulation of CD147 protein by transfection of shRNA expressing vector (Fig. 1B). Thus, SGC7901/shRNA2 cell clone was chosen for further experiments.

### CD147 silencing reduces the proliferation of SGC7901 cells

**Figure 2:**
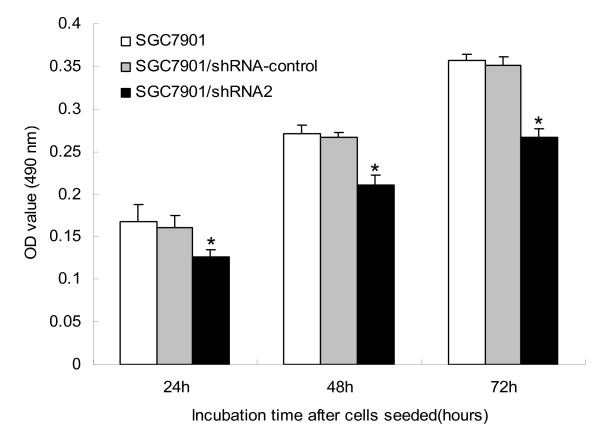
**Decrease in the proliferation potential of SGC7901 cells transfected with CD147 specific shRNA**. Gastric cancer cells (SGC7901, SGC7901/shRNA-control and SGC7901/shRNA2) seeded in 96-well microplates were cultured for 24, 48 and 72 h and their numbers were determined by absorbance. *p < 0.01 compared with SGC7901.

Next, we determined the proliferation of SGC7901, SGC7901/shRNA-control and SGC7901/shRNA2 respectively. As shown in Fig. 2, compared with SGC7901, the proliferation of SGC7901/shRNA2 was inhibited to 74.85% (p < 0.01), 77.86% (p < 0.01) and 74.79% (p < 0.01) at 24, 48 and 72 h, respectively. There was no significant difference between SGC7901/shRNA-control and SGC7901 (p > 0.05).

### CD147 silencing reduces MMP-2 and MMP-9 activities in SGC7901 cells

**Figure 3:**
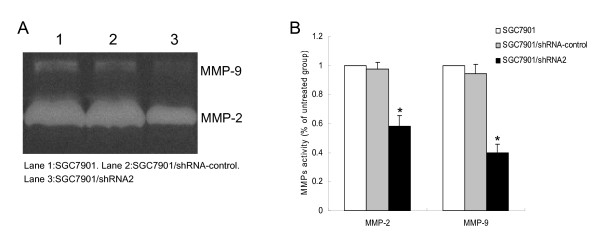
**Gelatin zymography analysis of MMP-2 and MMP-9 activity in SGC7901 cells**. Cells were incubated for 24 h and conditioned media were used for the measurement of MMP-2 and MMP-9 protein levels by gelatin zymography. (A) Photographs of the MMP-2 and MMP-9 bands, which are representative of three independent experiments, are shown. (B) Quantitative analysis of the bands. *p < 0.01 compared with SGC7901 and SGC7901/shRNA-control.

Since MMP-2 and MMP-9 play critical role in tumor cell invasion, we examined the effects of CD147 silencing on the enzyme activities of MMP-2 and MMP-9 using gelatin zymography. The gelatinolytic activities of both MMP-2 and MMP-9 were found to be reduced markedly in SGC7901/shRNA2 compared with SGC7901 and SGC7901/shRNA-control (p < 0.01) (Fig. 3). There was no significant difference between SGC7901/shRNA-control and SGC7901 (p > 0.05).

### CD147 silencing reduces the invasive ability of SGC7901 cells *in vitro*

**Figure 4:**
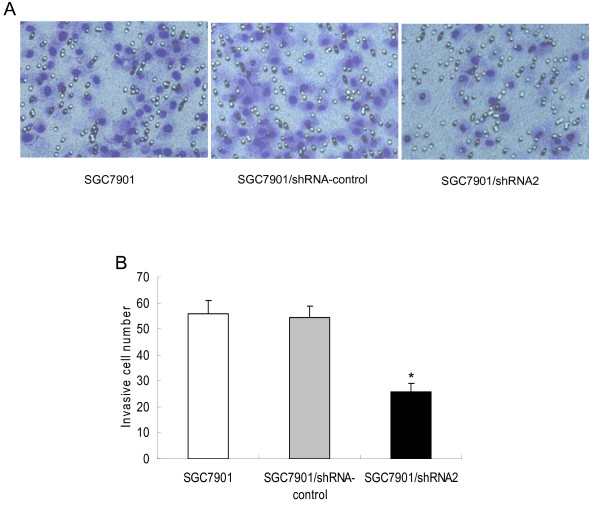
**Effects of CD147 specific shRNA on invasion of SGC7901 cells**. (A)Crystal violet staining results of the lower surface filters showed that the cells passed through the filter and attached to the lower side of the filter (400×). (B) The average number of cells that invaded through the filter was counted. The data were obstained from three independent experiments. *p < 0.01 compared with SGC7901 and SGC7901/shRNA-control.

To examine whether the down-regulation of CD147 in SGC7901 affected its invasive ability, we performed an *in vitro *Matrigel Transwell analysis. The results showed that SGC7901 and SGC7901/shRNA-control cells had a similar ability to pass through the Matrigel coated filter, because the numbers of invading cells were roughly equal (Fig 4). The number of SGC7901/shRNA2 cells (25.60 ± 3.28, p < 0.01) passing through the Matrigel was markedly lower than the numbers of SGC7901 (55.80 ± 5.03) and SGC7901/shRNA-control (54.40 ± 4.35) cells. There was no significant difference between SGC7901/shRNA-control and SGC7901 (p > 0.05).

### Silencing of CD147 in SGC7901 cells results in increased chemosensitivity to cisplatin

**Figure 5:**
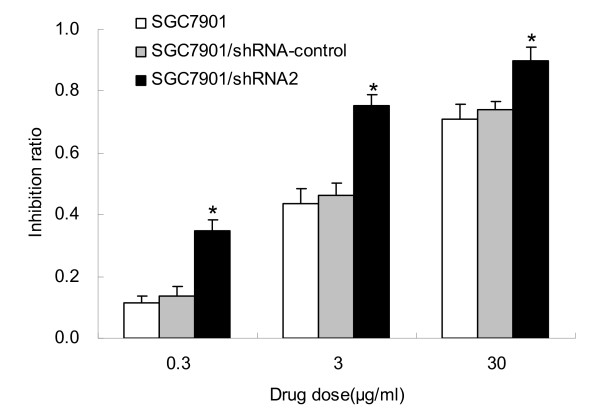
**Effects of CD147 specific shRNA on cisplatin sensitivity of SGC7901 cells**. SGC7901, SGC7901/shRNA-control and SGC7901/shRNA2 were treated with various concentrations of cisplatin. Cell vialility was determined by MTT assay. *p < 0.01 compared with SGC7901 and SGC7901/shRNA-control.

CD147 was found to be overexpressed in multidrug resistance tumor cells and could confer resistance to some anti-tumor drugs. We next investigated whether inhibition of CD147 by RNAi affected the sensitivity of SGC7901 cells to the anti-tumor drug cisplatin. As shown in Fig. 5, the inhibition rate in SGC7901/shRNA2 was markedly enhanced at all concentrations examined compared with SGC7901 and SGC7901/shRNA-control (p < 0.01). There was no significant difference between SGC7901/shRNA-control and SGC7901 (p > 0.05).

## Discussion

CD147, also designated EMMPRIN (extracellular matrix metalloproteinase inducer), is a cell surface glycoprotein which belongs to the immunoglobulin superfamily. As CD147 is highly expressed in most tumors and was shown to increase tumor invasion, most studies so far focuses on its role in cancer progression. However, its expression is not limited to tumor cells and was shown to be expressed in many cell types, including haematopoietic, epithelial, endothelial cells and leukocytes [14].

Gene silencing by RNA interference has emerged as a powerful method that is useful for the study of functional genomics [15]. Here, we successfully transfected two shRNAs targeting CD147 gene into human gastric cancer cell line SGC7901. Two stable cell clones SGC7901/shRNA1 and SGC7901/shRNA2 were obtained. CD147 expression was effectively inhibited at both mRNA and protein levels by shRNA2, while the shRNA1 was less efficient. These results indicated that shRNA targeting different sites of the same mRNA might be different in silencing efficiency.

We then examined the effect of CD147 silencing on the proliferation of SGC7901 cells. The proliferation potential of SGC7901/shRNA2 cells was suppressed compared with that of the control SGC7901 cells. Chen et al. and Jia et al. also found that down-regulation of CD147 inhibited the proliferation of human malignant melanoma cell line A375 and murine lymphoid neoplasm cell line P388D1, respectively [13,16]. It was found that CD147 and cyclophilin A (CypA) were both highly expressed in pancreatic cancer, and exogenous CypA promoted pancreatic cancer cell growth, which may be mediated through the interaction with its cellular receptor CD147 and the activation of ERK1/2 and p38 MAPKs [17].

Matrix metalloproteinases (MMPs), a family of zinc-dependent endopeptidases, play a crucial role in ECM degradation associated with cancer cell invasion, metastasis and angiogenesis [18]. Among members of the MMP family, MMP-2 (gelatinase-A) and MMP-9 (gelatinase-B) are particularly up-regulated in malignant tumors and contribute to the invasion and metastatic spread of cancer cells by degrading type IV collagen, a major component of the basement membrane [19]. The degree of MMP expression by stromal fibroblasts has been shown to be correlated with CD147 expression levels in a wide range of tumors [20]. CD147 was reported as the most constantly upregulated protein in metastatic cells, suggesting a central role in tumor progression and early metastasis [21]. Transfection of CD147 cDNA into human MDA-MB436 breast cancer cells resulted in an enhancement of tumor growth and an increase in metastatic incidences, both of which were directly correlated with high levels of tumor-derived MMP-2 and MMP-9 [22]. Among the MMPs induced by CD147, malignant progression has been most closely correlated with the expression of MMP-2 in several forms of cancer, and the increased levels of MMP-2 are typically indicative of poor prognostic outcome [23]. In our study, it was showed that downregulation of CD147 expression in human gastric cancer cells reduced the secretion of MMP-2 and MMP-9, thus inhibited the invasion ability of gastric cancer cells through the reconstituted basement membrane *in vitro*.

Multidrug resistance (MDR) is an important cause of treatment failure and mortality in gastric cancer patients. Overexpression of CD147 was observed in many MDR cancer cells [10]. CD147 plays a role in tumor MDR via different ways. CD147 was found to increase the expression of ATP-binding cassette (ABC) transporter families, such as *P*-glycoprotein (MDR1/ABCB1) [24,25]. CD147 was also shown to stimulate phosphoinositide 3-kinase/AKT cell survival signaling pathway, which is an anti-apoptotic pathway upregulated in most malignant cancer cells. The increase in anti-apoptotic signaling in turn leads to increased multidrug resistance. This effect of CD147 depends on stimulation of the production of hyaluronan, a pericellular polysaccharide [9,11]. The inhibition of CD147 expression via RNAi could increase the chemosensitivity to anti-tumor drugs in human ovarian cancer cell line and human oral squamous cell carcinoma cell line [26,27]. In the present study, silencing CD147 expression increased the chemosensitivity to cisplatin in human gastric cancer cell line SGC7901, suggesting CD147 is an adjuvant chemotherapy target for gastric cancer.

## Conclusions

This report showed that the silencing of CD147 by RNAi inhibited the proliferation and invasion of human gastric cancer cell line SGC7901 *in vitro *and increased its chemosensitivity to the anti-tumor drug cisplatin. Our findings suggested that CD147 might be a promising target for gastric cancer treatment.

## Abbreviations

ECM: extracellular matrix; MMPs: matrix metalloproteinases; RT-PCR: Reverse transcription-polymerase chain reaction.

## Competing interests

The authors declare that they have no competing interests.

## Authors' contributions

BW and YFX contributed equally to this work. BW, BSH, YQP and SKW designed research. BW, YFX, LRZ, CZ, LLQ performed research. BW and YQP analyzed data. BW wrote the paper. All authors read and approved the final manuscript.
